# The Prognostic Value of ctDNA and bTMB on Immune Checkpoint Inhibitors in Human Cancer

**DOI:** 10.3389/fonc.2021.706910

**Published:** 2021-10-01

**Authors:** Jiayan Wei, Jia Feng, Yiming Weng, Zexi Xu, Yao Jin, Peiwei Wang, Xue Cui, Peng Ruan, Ruijun Luo, Na Li, Min Peng

**Affiliations:** Department of Oncology, Renmin Hospital of Wuhan University, Wuhan, China

**Keywords:** ctDNA, bTMB, immune checkpoint inhibitor, prognosis, biomarker, meta-analysis

## Abstract

**Background:**

Circulating tumor DNA (ctDNA) levels and blood tumor mutation burden (bTMB) have a significant impact on the prognosis of tumor patients. However, their prognostic role in immune checkpoint inhibitors (ICIs) in cancer patients is still unclear.

**Methods:**

We used the Review Manager software (version 5.3) to perform a meta-analysis based on the published literature to explore the prognostic value of ctDNA and bTMB in patients receiving immunotherapy. We extracted the hazard ratios (HRs) of progression-free survival (PFS) and overall survival (OS) for each included study and their respective 95% confidence intervals (CIs) and *p*-values for analysis.

**Results:**

Thirteen studies were included in the meta-analysis. Higher ctDNA levels were significantly associated with shorter OS (HR = 3.35, 95%CI = 2.49–4.51, *p* < 0.00001) and PFS (HR = 3.28, 95%CI = 2.47–4.35, *p* < 0.00001). The results of ctDNA subgroup analysis showed that high posttreatment ctDNA levels significantly correlated with shorter OS in cancer patients receiving ICIs (HR = 5.09, 95%CI = 1.43–18.07, *p* = 0.01). Moreover, patients with ctDNA clearance had better OS (HR = 4.94, 95%CI = 2.96–8.26, *p* < 0.00001). Patients with high posttreatment ctDNA levels had shorter PFS (HR = 3.00, 95%CI = 2.02–4.46, *p* < 0.00001) and those with ctDNA clearance had longer PFS (HR = 4.61, 95%CI = 2.78–7.65, *p* < 0.00001). However, there was no statistically significant difference in the OS benefits between a high and a low bTMB after ICI therapy (HR = 0.68, 95%CI = 0.33–1.37, *p* = 0.28).

**Conclusions:**

The host immune system and tumor burden together determine whether cancer patients can benefit from ICI therapy. Our systematic review and meta-analysis revealed for the first time that the levels of pretreatment and posttreatment ctDNA and the clearance of ctDNA can independently be used as prognostic factors for antitumor immunotherapy, while bTMB cannot. In conclusion, ctDNA levels have great potential as an assistant tool for radiological assessments to make clinical therapeutic decisions. The prognostic utility of bTMB still requires further exploration.

## Introduction

Circulating tumor DNA (ctDNA), a component of cell-free DNA (cfDNA), is released from apoptotic or necrotic tumor cells (1). ctDNA can be measured by polymerase chain reaction (PCR) and next-generation sequencing (NGS) technology, and it is expected to be a new indicator for evaluating tumor burden and treatment response (2). Blood-based tumor mutation burden (bTMB) is the number of mutations per megabase (Mut/Mb) detected in the ctDNA sequencing region and is considered to be a neoantigen load marker that stimulates the immune response of T cells (3). In the past few decades, immune checkpoint inhibitors (ICIs) have been widely used and have shown remarkable effects in a variety of solid tumors, such as non-small cell lung cancer, melanoma, and renal cell carcinoma (4, 5). However, the objective response rate (ORR) was lower than 30% in unselected patients (6), highlighting the need for new biomarkers to identify patients who are more likely to benefit from ICI therapy. Tissue TMB (tTMB) has been used in multiple studies as a biomarker to predict the response to immunotherapy. However, owing to its invasiveness and organizational spatial heterogeneity, operable, easily accessible, and real-time ctDNA and bTMB have attracted more attention.

Several studies have focused on the prognostic impact of ctDNA and bTMB in patients receiving immunotherapy (7–9). However, most of them are characterized by small sample sizes and low universality. Therefore, we conducted a systematic review and meta-analysis on this topic.

## Materials and Methods

### Search Strategy and Study Selection

Relevant published literature was searched for using MEDLINE (PubMed) and EMBASE. The following search terms were used: ctDNA OR circulating biomarker AND immune checkpoint AND cancer NOT review, ctDNA AND predictive AND cancer AND immunotherapy. The last search was updated on August 28, 2021.

The included studies met the following criteria: 1) cohort studies or clinical trials that use ICIs for treatment and ctDNA or bTMB to predict efficacy; 2) the prognostic value of ctDNA or bTMB in cancer patients who had received immunotherapy was investigated; 3) hazard ratios (HRs) of overall survival (OS) and progression-free survival (PFS), as well as their 95% CIs and *p*-values, or sufficient data to calculate them.

The exclusion criteria were as follows: 1) reviews, case reports, meeting abstracts, letters, expert opinions, and animal studies; and 2) no English translation of the study.

### Data Extraction

Data were extracted from the included studies. The following pieces of information were extracted from each study: author name, year of publication, tumor type, study type, blood biomarker type, timing of biomarker, biomarker detection method, cutoff point of blood biomarker, type of ICI used, type of outcome, and results (HRs and 95% CIs).

### Quality Assessment

The risk bias evaluation tool (Cochrane Handbook for Systematic Reviews of Interventions) was used to evaluate the quality of the included studies. Seven evaluation items were used to examine the quality of the research: random sequence generation, allocation concealment, blinding of participants and personnel, blinding of outcome assessment, incomplete outcome data, selective reporting, and other sources of bias.

### Statistical Analysis

We used the Review Manager software (version 5.3) to analyze the prognostic effects of ctDNA and bTMB in tumor patients receiving ICI therapy. The HRs of PFS and OS and their 95% CIs were used to calculate the pooled estimates of the meta-analysis. Statistical significance was set at *p* < 0.05. The heterogeneity of each study was tested using the Higgins *I* ^2^ statistic. If *I* ^2^ was greater than 50%, it was considered that there was significant heterogeneity between the studies, so the random effects model was used; otherwise, when there was no significant heterogeneity (*I* ^2^ < 50%), the fixed effects model was selected. There is no absolute definition of ctDNA or bTMB. The cutoff points for ctDNA and bTMB are not uniform because the studies we included used different techniques to detect biomarkers. To better analyze the data, we defined those biomarkers with values greater than the cutoff points and were detectable, positive, and unclear as high levels of ctDNA or bTMB and, conversely, as low levels of ctDNA or bTMB.

## Results

### Study Characteristics

**Figure 1:**
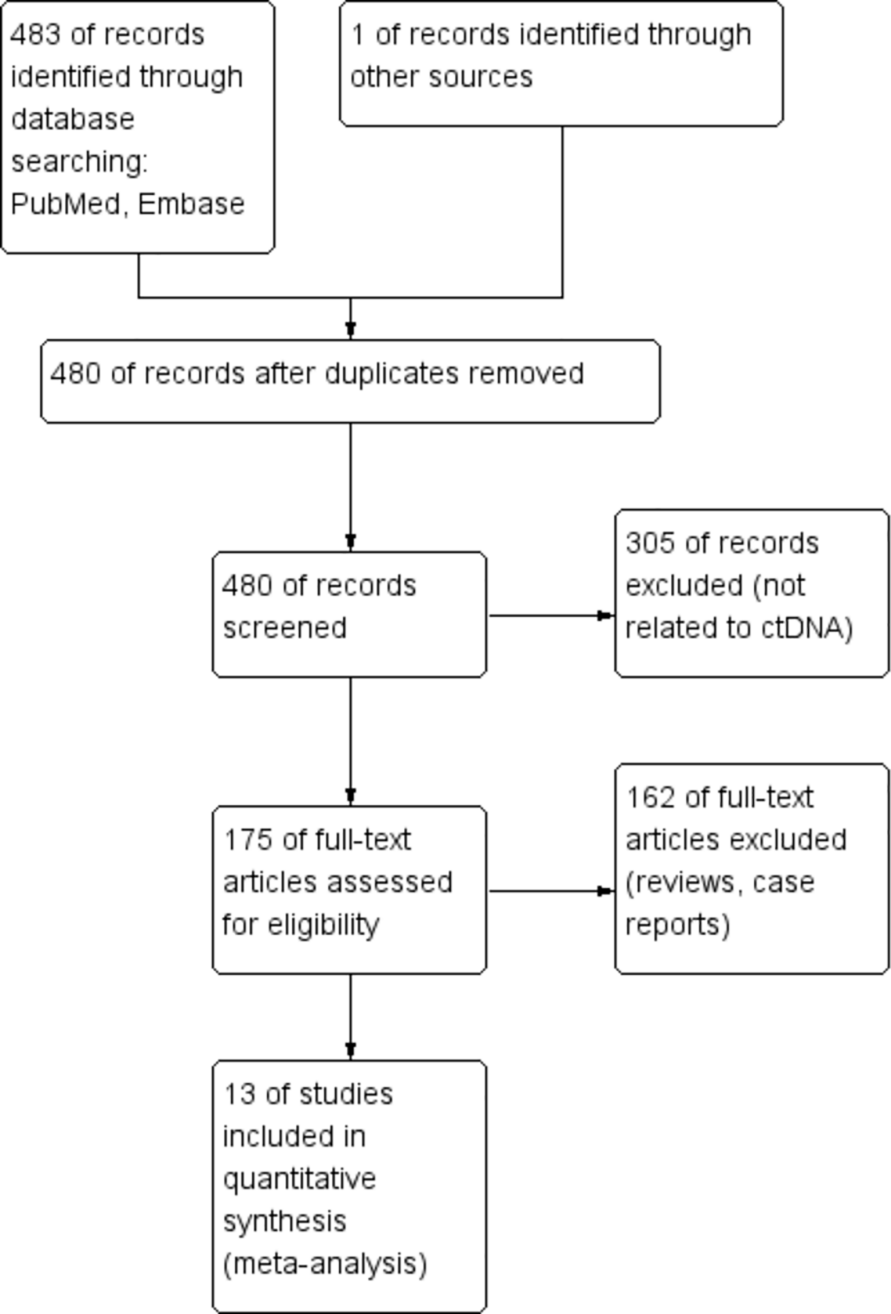
Enrollment process of the included studies. The processes of identification, screening, eligibility, and inclusion are shown.

A total of 484 articles were retrieved through a database search. Using the exclusion criteria listed above, we removed 4 duplicate articles, 305 articles not related to ctDNA and bTMB, and 162 articles from non-clinical studies. Thirteen articles were finally included in our meta-analysis. The enrollment process of this study is shown in **Figure 1**. Among the 13 included studies, regarding tumor types, four studies were on non-small cell lung cancer (NSCLC), four were on melanoma, one was on colorectal cancer, one was on biliary tract cancer, and one was on urothelial carcinoma; the remaining two were studies on a mixture of different cancers. **Table 1** summarizes the characteristics of the 13 included studies.

**Table 1:** Characteristics of the included studies.

Authors	Year	Cancer type	Study type	Biomarker type	Timing of biomarker	Biomarker detection method	Cutoff point	ICI	Outcome of interest	Results
Chen et al.	2020	Colorectal cancer	Prospective	bTMB	Pretreatment	NGS	≥28 *vs*. <28 vts/Mb	Tremelimumab, durvalumab	OS	HR = 0.34, 90%CI = 0.18–0.63, *p* = 0.004
Lee et al.	2020	Melanoma	Prospective	ctDNA	Pretreatment	PCR	Undetectable *vs*. detectable	Pembrolizumab, nivolumab, ipilimumab	OS	HR = 0.51, 95%CI = 0.28–0.94, *p* = 0.03
Wang et al.	2020	NSCLC	Prospective	bTMB	Not mentioned	NGS	≥6 *vs*.<6 vts/Mb	Atezolizumab, nivolumab, pembrolizumab, tislelizumab, toripalimab	OS	HR = 0.92, 95%CI = 0.46–1.82, *p* = 0.80
Wang et al.	2020	NSCLC	Prospective	MSAF (ctDNA)	Not mentioned	NGS	Top 25% *vs*. bottom 75%	Atezolizumab, nivolumab, pembrolizumab, tislelizumab, toripalimab	OS	HR = 2.72, 95%CI = 1.33–5.59, *p* = 0.005
Chen et al.	2020	Biliary tract cancer	Prospective	ctDNA	Posttreatment	NGS	Positive *vs*. negative	Camrelizumab	OS and PFS	OS: HR = 1.77, 95%CI = 0.78–3.99, *p* = 0.16
										PFS: HR = 2.83, 95%CI = 1.27–6.28, *p* = 0.007
Chen et al	2020	Biliary tract cancer	Prospective	bTMB	Not mentioned	NGS	Top 25% *vs*. bottom 75%	Camrelizumab	OS and PFS	OS: HR = 1.05, 95%CI = 0.43–2.54, *p* = 0.92
										PFS: HR = 2.57, 95%CI = 1.08–6.12, *p* = 0.03
Pedersen et al.	2020	Melanoma	Prospective	ctDNA	Posttreatment	PCR	Detectable *vs*. undetectable	Pembrolizumab, nivolumab, ipilimumab	PFS	HR = 7.89, 95%CI = 1.40–44.6, *p* = 0.019
Marsavela et al.	2020	Melanoma	Prospective	ctDNA	Pretreatment	PCR	≤20 *vs*. >20 copies/ml	Nivolumab, pembrolizumab, ipilimumab	PFS	HR = 0.42, 95%CI = 0.22–0.83, *p* = 0.006
Anagnostou et al.	2020	NSCLC	Prospective	ctDNA	Clearance	NGS	No complete reduction *vs*. complete reduction	Unclear	OS and PFS	OS: HR = 6.91, 95%CI = 1.37–34.97, *p* = 0.02
										PFS: HR = 5.36, 95%CI = 1.57–18.35, *p* = 0.007
Goldberg et al.	2018	NSCLC	Prospective	ctDNA	Clearance	NGS	>50% *vs*. ≤50% decrease in mutant allele fraction from baseline	Unclear	OS and PFS	OS: HR = 0.17, 95%CI = 0.05–0.62, *p* = 0.007
										PFS: HR = 0.29, 95%CI = 0.09–0.89, *p* = 0.03
Cabel et al.	2017	NSCLC, etc.	Prospective	ctDNA	Posttreatment	NGS	Detectable *vs*. undetectable	Nivolumab, pembrolizumab	OS and PFS	OS: HR = 15, 95%CI = 2.5–94.9, *p* = 0.004
										PFS: HR = 10.2, 95%CI = 2.5–41, *p* < 0.001
Herbreteau et al.	2021	Melanoma	Prospective	ctDNA	Clearance	PCR	Increase *vs*. decrease	Nivolumab/nivolumab + ipilimumab	OS and PFS	OS: HR = 7.49, 95%CI = 2.59–24.10, *p* = 0.0002
										PFS: HR = 12.74, 95%CI = 3.81–53.25, *p* < 0.0001
Ricciuti et al.	2021	NSCLC	Retrospective	ctDNA	Clearance	NGS	Decrease *vs*. increase	Pembrolizumab	OS and PFS	OS: HR = 0.34, 95%CI = 0.15–0.75, *p* = 0.008
										PFS: HR = 0.29, 95%CI = 0.14–0.60, *p* = 0.0007
Zhang et al.	2020	Advanced cancers	Prospective	ctDNA	Posttreatment	Not mentioned	Below median *vs*. above median	Durvalumab ± tremelimumab	OS and PFS	HR = 0.13, 95%CI = 0.05–0.34
										HR = 0.41, 95%CI = 0.25–0.68
Powles et al.	2021	Urothelial carcinoma	Prospective	ctDNA	Clearance	PCR	Clear *vs*. not clear	Atezolizumab	OS	HR = 0.14, 95%CI = 0.03–0.59

vts/Mb, variations per megabase; ctDNA, circulating tumor DNA; bTMB, blood tumor mutation burden; ICI, immune checkpoint inhibitor; HR, hazard ratio; NSCLC, non-small-cell lung cancer; MSAF, maximum somatic allele frequency; NGS, next-generation sequencing; PCR, polymerase chain reaction; OS, overall survival; PFS, progression-free survival.

### Risk of Bias

**Figure 2:**
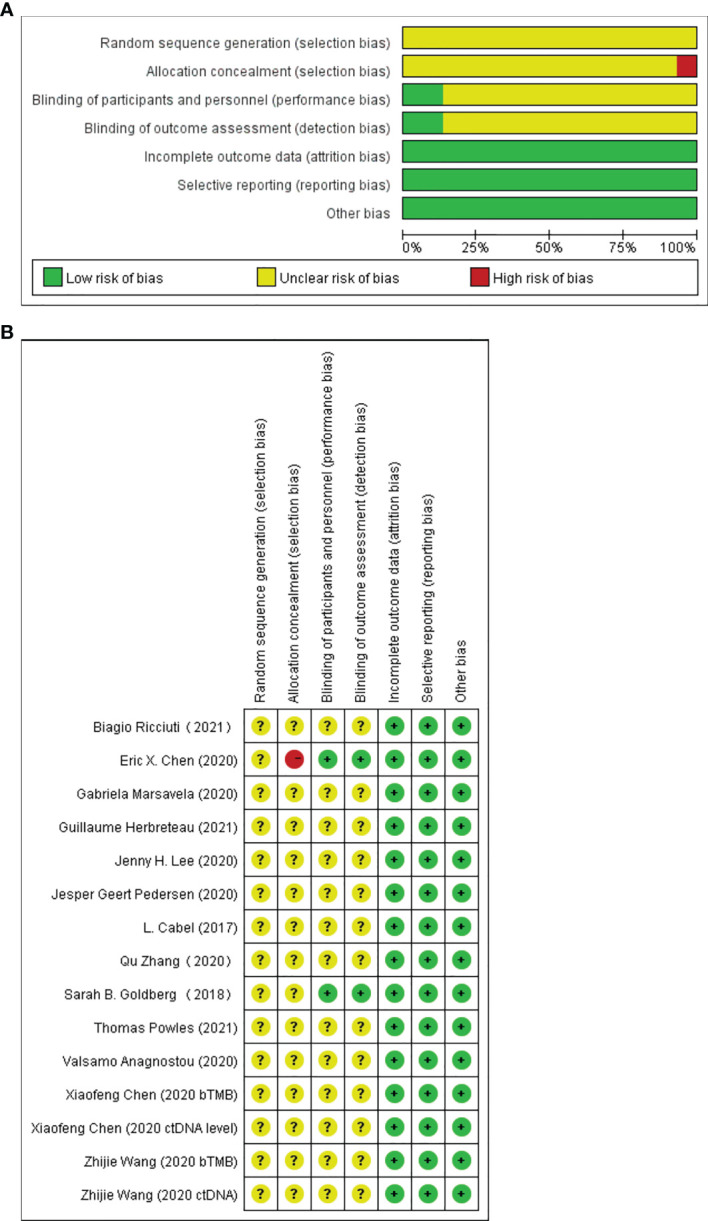
Assessment of risk of bias at the study level. **(A)** Risk of bias graph: review authors’ judgments of each risk of bias item presented as percentages across all included full report studies. **(B)** Risk of bias summary: review authors’ judgments of each risk of bias item.

**Figure 3:**
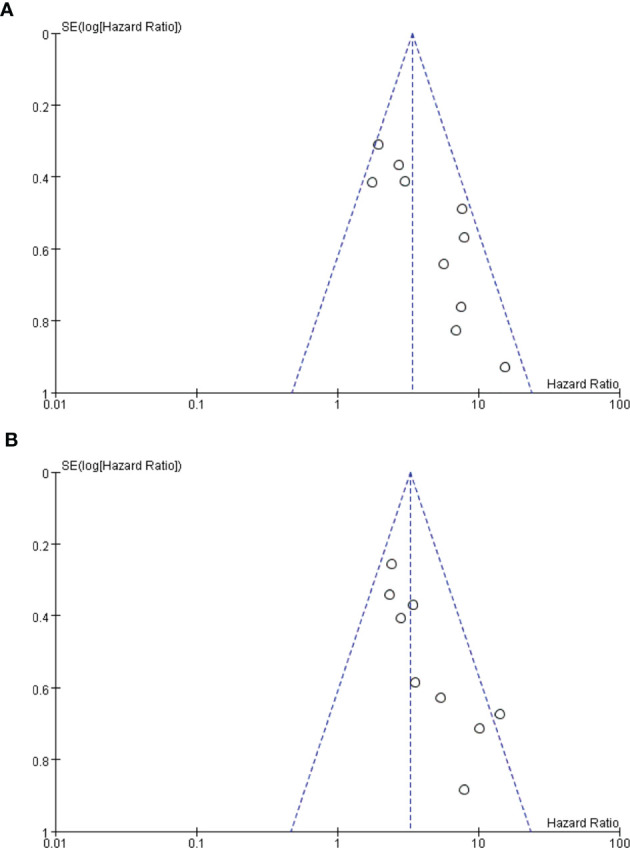
**(A, B)** Funnel plots. Funnel plot analysis on potential publication bias for overall survival (OS) **(A)** and progression-free survival (PFS) **(B)**.

Twelve of the 13 included studies were prospective cohort studies and only one was a retrospective cohort study, so the overall risk of bias was relatively low. **Figures 2A, B** summarize the risk bias of all the included studies. **Figures 3A, B** display the funnel plots showing no significant publication bias affecting the HRs of OS and PFS on ctDNA.

### Outcomes of Included Studies

#### Relationship Between ctDNA Levels and Response to Immunotherapy

**Figure 4:**
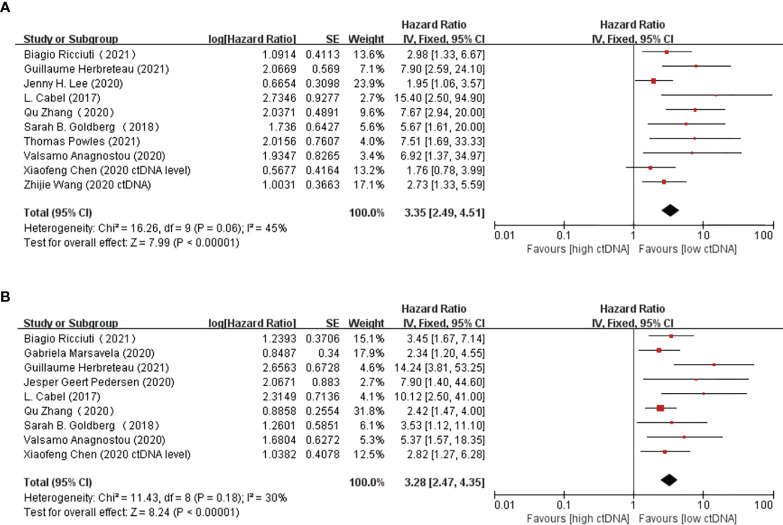
**(A, B)** Forest plots of the fixed effects meta-analysis on the efficacy of circulating tumor DNA (ctDNA) for overall survival (OS) **(A)** and for progression-free survival (PFS) **(B)**.

**Figure 5:**
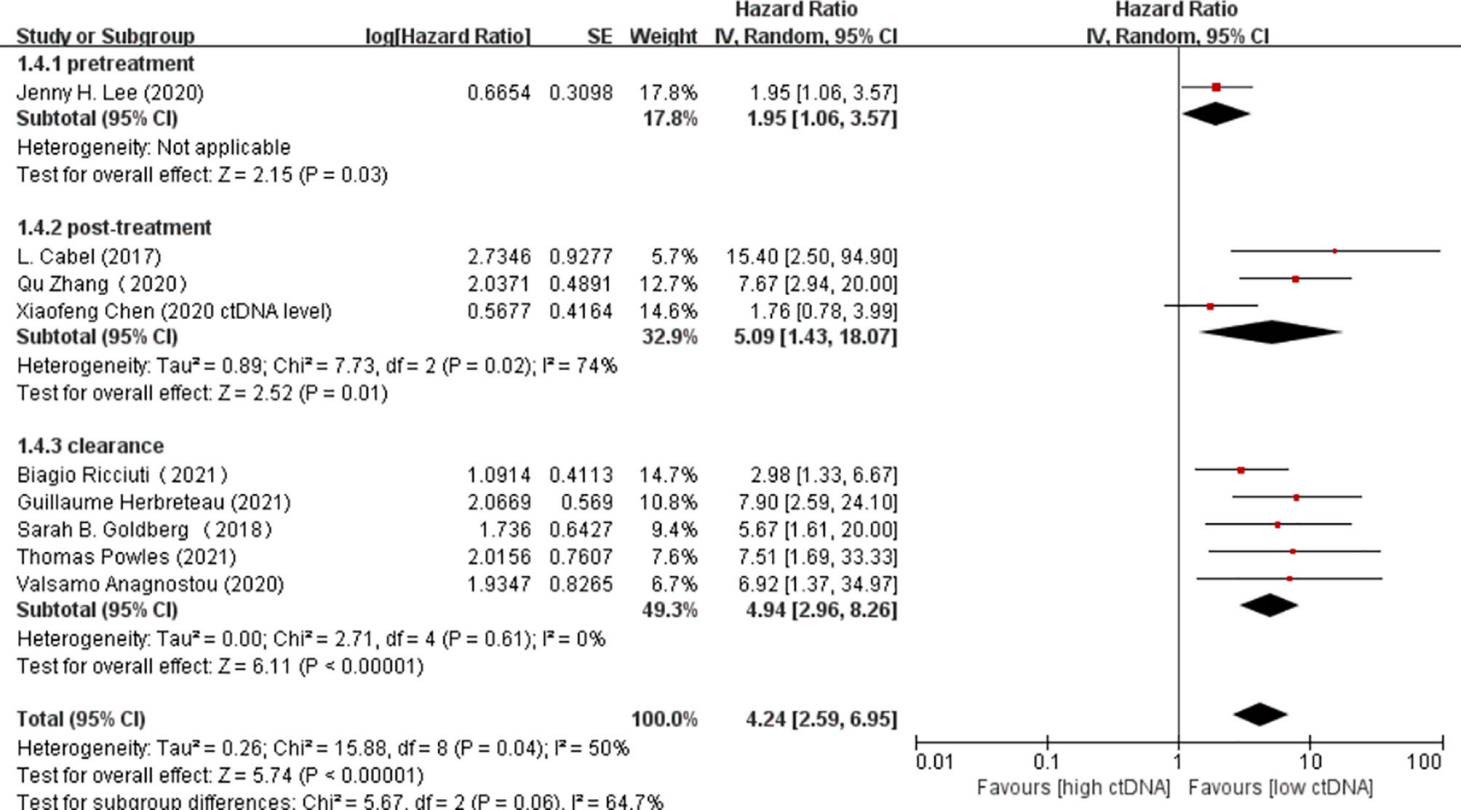
Forest plot of the random effects meta-analysis on the efficacy of circulating DNA (ctDNA) for overall survival (OS) at different time points.

**Figure 6:**
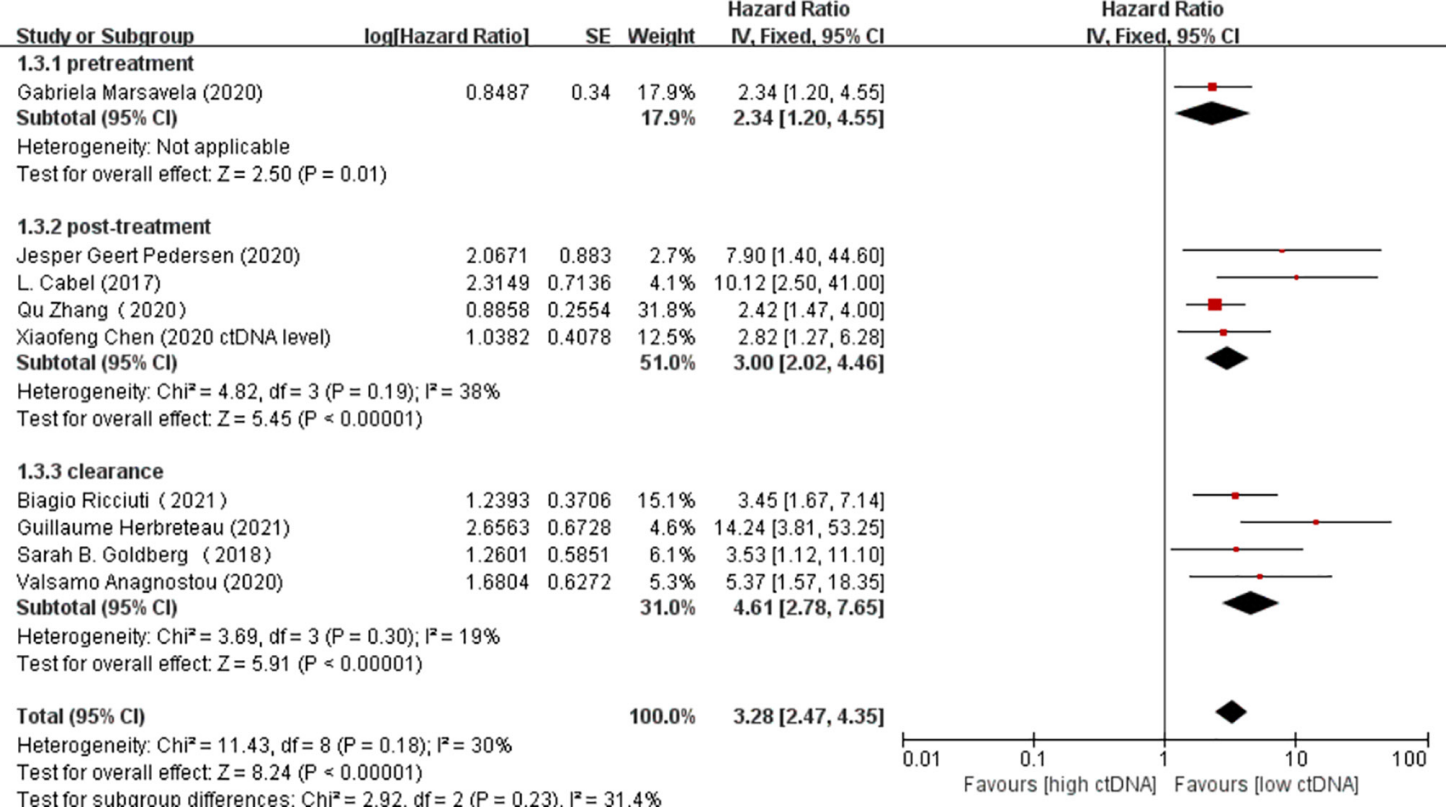
Forest plot of the fixed effects meta-analysis on the efficacy of circulating DNA (ctDNA) for progression-free survival (PFS) at different time points.

Overall, there were 10 studies on the prognostic value of ctDNA levels in the OS of patients receiving immunotherapy. Elevated ctDNA levels were associated with shorter OS (HR = 3.35, 95% CI = 2.49–4.51, *p* < 0.00001) (**Figure 4A**). A total of nine studies were eligible for inclusion in the meta-analysis regarding the prognostic value of ctDNA levels in the PFS of patients receiving ICI therapy. A statistically significant poorer PFS was also observed in patients with higher ctDNA levels, with a pooled HR of 3.28 (95%CI = 2.47–4.35, *p* < 0.00001) (**Figure 4B**). In the subgroup analysis of the different timings of biomarkers, high posttreatment ctDNA levels significantly correlated with shorter OS in cancer patients receiving ICIs (HR = 5.09, 95%CI = 1.43–18.07, *p* = 0.01). In addition, patients without ctDNA clearance had worse OS (HR = 4.94, 95%CI = 2.96–8.26, *p* < 0.00001). There was only one study on the relationship between the pretreatment ctDNA levels and OS, and the results showed that high pretreatment ctDNA levels were correlated with worse overall survival (HR = 1.95, 95%CI = 1.06–3.57, *p* = 0.03) (**Figure 5**). As for PFS, patients with high posttreatment ctDNA levels had shorter PFS (HR = 3.00, 95%CI = 2.02–4.46, *p* < 0.00001). Similarly, patients with ctDNA clearance had longer PFS (HR = 4.61, 95%CI = 2.78–7.65, *p* < 0.00001). In addition, high levels of pretreatment ctDNA were significantly correlated with shorter PFS (HR = 2.34, 95%CI = 1.20–4.55, *p* = 0.01) (**Figure 6**).

#### Relationship Between bTMB and Response to Immunotherapy

**Figure 7:**
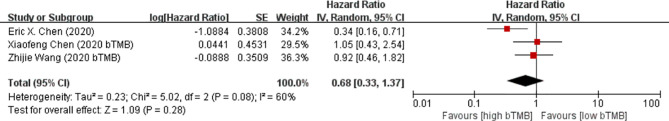
Forest plot of the random effects meta-analysis on the efficacy of blood tumor mutation burden (bTMB) for overall survival (OS).

There was only one study with PFS as an outcome indicator. Estimation of the prognostic value of bTMB in the PFS of patients receiving ICI therapy revealed that a high bTMB was significantly associated with shorter PFS (HR = 2.57, 95%CI = 1.08–6.12, *p* = 0.03). There were a total of three studies on the prognostic value of bTMB in the OS of cancer patients receiving immunotherapy. The pooled results showed that there was no statistically significant difference in the OS benefits between a higher and a lower bTMB (HR = 0.68, 95%CI = 0.33–1.37, *p* = 0.28) (**Figure 7**).

### Heterogeneity

In the analysis of the prognostic effect of ctDNA in patients receiving immunotherapy, no significant heterogeneity was observed in the outcomes of PFS and OS (*I* ^2^ = 30%, *p* < 0.00001; *I* ^2^ = 45%, *p* < 0.00001); thus, both were analyzed with the fixed effects models. The heterogeneity between the studies on bTMB was greater than 50% (*I* ^2^ = 60%), so the random effects model was selected.

## Discussion

The efficacy of ICIs mainly depends on the tumor burden and the immune system of the host (10–12). At present, the main tools used to evaluate disease burden and the host immune status are radiologic assessments (CT and MRI) and tTMB (13–17), but they all have their own limitations. The clinical decision to continue or suspend ICI therapy is usually guided by continuous radiographic observations of changes in the tumor. However, CT and MRI are unable to identify patients who can achieve benefits early because tumors usually shrink slowly (18). In addition, radiographs often fail to identify whether transient tumor enlargements come from true disease progression or pseudoprogression, the latter referring to immune cell infiltration (18–20). Relevant evidence has shown that the existence of ctDNA occurs earlier than the recurrence of radiographic imaging, and it dynamically changes with the patient’s response to treatment (21). As a prognostic factor of the host immune status, tTMB is also not completely satisfactory. Firstly, the measurement of tTMB requires tumor biopsy material, which may cause trauma and bleeding. Secondly, not all cancer patients meet the criteria for tissue biopsy (22). Thirdly, tTMB can only reflect the mutation burden of local tumor tissues and does not focus on the whole body (23). Finally, tTMB is unable to dynamically monitor tumor burden in real time. In order to more accurately identify patients who are most likely to benefit from immunotherapy, new biomarkers are needed to compensate for the lack of the evaluation tools mentioned above. ctDNA and bTMB are expected to become new biomarkers, but their exact prognostic roles in ICI therapy remain to be clarified. To the best of our knowledge, this is the first systematic review and meta-analysis on the prognostic impact of ctDNA and bTMB in patients undergoing immunotherapy.

Some studies claimed that a higher bTMB indicated better prognosis, which means longer PFS and OS in patients receiving immunotherapy (24, 25), while others hold the opposite opinion (26). The pooled results of our meta-analysis revealed that higher ctDNA levels resulted in shorter PFS (HR = 3.28, 95%CI = 2.47–4.35, *p* < 0.00001) and OS (HR = 3.35, 95%CI = 2.49–4.51, *p* < 0.00001). In the subgroup analysis of biomarkers at different time points, patients with high levels of pretreatment or posttreatment ctDNA and patients without ctDNA clearance during treatment all had worse prognosis (PFS and OS) in immunotherapy. Regarding bTMB, no statistically significant difference was observed between a high and a low bTMB in OS prognosis (HR = 0.68, 95%CI = 0.33–1.37, *p* = 0.28).

ctDNA is a single- or double-stranded DNA released into the blood by tumor cells. The proportion of ctDNA in cfDNA ranges widely, and it is determined by the synthesis of tumor location, phenotype, and differentiation degree (27). Therefore, ctDNA can reflect the burden of tumors and carry the original tumor mutations (28). Theoretically, a higher ctDNA level reveals a greater tumor burden, resulting in a poorer prognosis. Zhao et al. (29) also observed that, in liver cancer, higher ctDNA levels were more associated with larger tumor volumes than was alpha-fetoprotein (AFP). This finding was consistent with the results of our meta-analysis.

Synonymous variation, non-synonymous variation, and variation of unknown significance (VUS) are the three methods used to calculate bTMB (3). New somatic mutations in tumor cells result in new antigen expression, and the production of tumor-specific antigens is an important prerequisite for T cells to recognize tumors (30, 31). Moreover, neoantigens produced by mutations in tumor somatic cells have been confirmed to activate the immune response of T cells (32). Previous studies have demonstrated that a higher tTMB is associated with longer OS and PFS in patients receiving immunotherapy (33–35). The feasibility and accuracy of bTMB measured from blood samples based on ctDNA and the positive association between bTMB and TMB in tumor tissues have been confirmed (36, 37). Therefore, in theory, bTMB also has prognostic value in patients receiving ICIs, and a higher bTMB corresponds to better survival. However, the pooled results of our meta-analysis revealed no statistically significant relationship between a higher bTMB and better OS. Why bTMB cannot be a prognostic factor in patients receiving ICIs will be explained in the following. The detection method for bTMB inevitably leads to the following results: ctDNA levels have an important impact on the abundance of bTMB. In this way, a higher bTMB may be accompanied by higher ctDNA levels, and the latter is closely correlated with worse prognosis. As a consequence, a higher bTMB does not necessarily reveal longer OS and PFS; likewise, a lower bTMB is not necessarily related to shorter OS and PFS. In conclusion, some problems remain to be overcome before the clinical implementation of bTMB. To effectively determine the prognostic value of bTMB in cancer patients undergoing immunotherapy, the integration of bTMB and other blood biomarkers in the future may be required.

Our meta-analysis explored the prognostic value of high or low ctDNA and bTMB in patients receiving immunotherapy, but did not address the predictive effect of ctDNA or bTMB on the outcome of immunotherapy. The results of the trial, published in Nature by Powles et al., revealed that the ctDNA-positive patients in the atezolizumab group had better prognosis than those in the observation group, suggesting that ctDNA may be a predictor of the efficacy of ICIs. This conclusion is helpful in the clinical decision-making of clinicians. For patients with positive ctDNA after tumor surgery, the use of ICIs may be an option to improve survival. However, there are limited studies on the predictive indicators of the efficacy of immunotherapy, and this conclusion needs to be confirmed by more data in future studies.

Our study had certain limitations. Firstly, since the detection technology of ctDNA and bTMB in blood is still in the initial stages of development, there will be more or less inconsistencies between the measured values and the true values, which is also the main reason for the different cutoff points of ctDNA and bTMB in all the studies included in our meta-analysis. Therefore, the stability of our meta-analysis results was affected. Secondly, the number of studies included in the meta-analysis was relatively small, especially the number of studies on bTMB. Thirdly, in addition to the different cutoff points of the biomarkers that affect the results of the analysis, there are other factors that will cause heterogeneity in the meta-analysis results and affect the authenticity and reliability of the final results. Although we have performed a subgroup analysis on the prognostic value of ctDNA in patients receiving immunotherapy at different time points, the details of each study in each subgroup were diverse. For example, although they were all studies on the prognostic value of posttreatment ctDNA levels in patients receiving ICIs, some studies focused on ctDNA at 6–8 weeks after immunotherapy while others explored ctDNA at 8–10 weeks after immunotherapy. In addition, for studies on the prognostic impact of ctDNA clearance, the definition and the standard of ctDNA clearance were different. Finally, the detection methods for ctDNA and bTMB used by the studies included in our meta-analysis were not uniform (PCR and NGS, respectively), which would also impact the results of the analysis. This requires the continuous updating and improvement of the detection methods for these two biomarkers in the future.

## Conclusion

In the past, ctDNA and bTMB have received increased attention in the field of targeted therapy and chemo/radiotherapy (38–41), but there has been no consensus regarding their prognostic role in patients receiving ICIs. Our meta-analysis results demonstrated that the levels and the clearance of ctDNA can be used as independent prognostic factors for immunotherapy, while the prognostic impact of bTMB in cancer patients undergoing immunotherapy is worth further discussion and exploration.

Monitoring the ctDNA levels for ICI therapy has the following advantages: it can be performed in real time, is noninvasive, and is ultrasensitive. Therefore, it can be a good prognostic factor for immunotherapy in patients with cancer. Monitoring ctDNA can be used as an important supplement to conventional imaging and help in making timely therapeutic management decisions. Due to the limitations of the current detection technology and standards, bTMB cannot be directly used as a prognostic factor to effectively predict the survival of patients undergoing treatment with ICIs.

## Author Contributions

YW, NL, and MP conceptualized the study. PR, YJ, and ZX contributed to the methodology. JW helped with software. RL did the formal analysis. JW, JF, PW, and XC prepared the original draft. YW reviewed and edited the manuscript. All authors contributed to the article and approved the submitted version.

## Funding

This work was supported by grants from the National Natural Science Foundation of China (81770169), National Natural Science Foundation of China (81802980) and National Natural Science Foundation of China (81102024).

## Conflict of Interest

The authors declare that the research was conducted in the absence of any commercial or financial relationships that could be construed as a potential conflict of interest.

## Publisher’s Note

All claims expressed in this article are solely those of the authors and do not necessarily represent those of their affiliated organizations, or those of the publisher, the editors and the reviewers. Any product that may be evaluated in this article, or claim that may be made by its manufacturer, is not guaranteed or endorsed by the publisher.
